# Perspectives of Information Access in the Informed Consent Process for Clinical Research Participation in Australia

**DOI:** 10.1002/eahr.70011

**Published:** 2026-05-04

**Authors:** Fleur O'Hare, Lauren N. Ayton, David Foran, Camille Paynter, Michelle Gallaher, Kelly Schulz, Myra B. McGuinness, Lauren Barina, Steven Y. C. Tong, Tessa Saunders

**Affiliations:** ^1^ Lead researcher and consumer involvement & advocacy lead in the Department of Optometry and Vision Sciences at the Melbourne School of Health Sciences at the University of Melbourne; ^2^ Research fellow in the Department of Optometry and Vision Sciences at the Melbourne School of Health Sciences at the University of Melbourne; ^3^ Access and inclusion specialist, is a consumer research partner at Equalus Advisory; ^4^ Clinical researcher in the Department of Audiology and Speech Pathology at the Melbourne School of Health Sciences at the University of Melbourne; ^5^ Research partner at Cerulea Clinical Trials; ^6^ Consumer research partner at Knowable Me; ^7^ Biostatistician at the Centre for Eye Research Australia; ^8^ Global clinical trial manager in the Department of Infectious Diseases at the Peter Doherty Institute for Infection and Immunity at the University of Melbourne; ^9^ Professorial fellow and clinical researcher at the Victorian Infectious Diseases Service at the Royal Melbourne Hospital at the Peter Doherty Institute for Infection and Immunity; ^10^ Research fellow in the Evaluation and Implementation Science Unit at the Melbourne School of Population and Global Health at the University of Melbourne

**Keywords:** informed consent, human research ethics, research participation, clinical research participant, good clinical practice, inclusion, equity, access

## Abstract

Access to communication and information during the informed consent process for clinical research is essential for empowered decision‐making. To build awareness of accessibility practices, this study aimed to explore current enablers and barriers experienced by potential research participants during informed consent procedures. A multimodal survey, capturing quantitative and qualitative data, was distributed through industry networks in Australia. The survey was open to people who had been involved in an informed consent process for a clinical research study. We collected survey data from August 2024 to January 2025. Quantitative data were reported descriptively, while qualitative data were analyzed using content analysis, with barriers and facilitators mapped to the Theoretical Domains Framework. Half of the respondents (52%) disclosed having an information access challenge impacted by vision, intersectional disability, or neurodiversity. Five themes around information access were identified: communication, information quality, assistive technology, trusted supports, and information intervals. Our research highlights some suggested actions that can support researchers and research organizations in providing accessible communication and information formats, thereby enhancing equity and inclusion in informed consent practices.

The process of obtaining informed consent is akin to an “information highway” in clinical research.^1^ Ideally the highway of information is shared in both directions. For both the researcher and potential research participants, shared information is empowering. Enabling autonomy and agency in the informed consent process is key to this. For someone to be truly informed about research they must be engaged in a process that meets their communication and information needs. Communication and information provision require proactive steps to be taken by the researcher to enhance information readability and understanding, and to remove barriers to accessing information. Therefore, it is the responsibility of research organizations and researchers to redress or reduce health inequities that may arise from inaccessible communication and information provision.

Accessible informed consent practices are fundamental to conducting ethical clinical research. An informed consent process must involve sharing information with potential participants to elevate informed decision‐making.^2^ Recent updates to industry‐based Good Clinical Practice (GCP)^3^ guidelines provide a flexible framework for clinical trial conduct in recognition of individual communication and information needs. For example, the International Council for Harmonization GCP Guideline suggests that information provided during the informed consent process should be clear, concise and easy to understand (recommendations 2.2 and 2.8.6).^3^ Furthermore, information should be presented in plain language and “may be presented in varied formats for example text, videos and other interactive methods” (recommendation 2.8.1). The aim of these flexible methods is to optimize engagement with individuals during the informed consent process. Yet despite this guidance, there remains a plethora of complex information sheets and consent forms being routinely used in practice.^4^, ^5^, ^6^, ^7^, ^8^ Numerous investigations into informed consent practices have stressed the importance of simplified formats.^9^, ^10^, ^11^ This is in parallel with a growing number of studies showing that effective adaptation and tailoring of the informed consent process to meet the needs of the priority group enhances understanding,^12^ acceptability^13^ and retention in the study over the long term.^14^ From the perspective of potential and actual participants, it has been reported that a layered approach to information provision, where the person may choose how much information to receive, and in what format, enhances empowerment in the informed consent process.^15^

In Australia, 44% of the population has low health literacy.^16^ Additionally, one in five people have a disability that may impact their ability to receive and relay communication and information,^17^ with similar rates worldwide.^18^ Accessibility practices should be standard practice and available to all, regardless of their identity or background. Several studies have explored the barriers and enablers within informed consent practices in Australia.^7^, ^15^ This is similar to the research conducted by the CT:IQ working group, a national group of consumers and experts working in varied roles across the clinical trial sector in Australia.^19^ The CT:IQ group's body of research has included developing a Participant Information and Consent Form (PICF) template, called the InFORMed template,^15^ which is currently being evaluated in practice.^20^

The InFORMed template is not yet widely used but heralds a new era using a participant‐centered template that has been codesigned with consumers and offers plain language guidance. Plain language means using simpler word choices (semantics), simplified sentence structure (syntax), the use of shorter sentences, using active voice and present tense, and focusing on what potential research participants need to know to understand study requirements. From the perspective of potential participants, it is crucial to gain a complete understanding of the direct impacts to them from participating in clinical research such as time commitment, and possible risks and benefits. Evaluating opportunities for inclusive consent practices helps broaden the understanding of community needs.

Our study aimed to explore the main barriers and enablers within informed consent processes from the perspective of individuals who had been involved in an informed consent process for research participation. We wanted to investigate what was working well and what was not working well from a consumer lens in the Australian research sector. This approach aligns with implementation science methodologies, such as the Theoretical Domains Framework (TDF), that help to categorize identified barriers and enablers.^21^, ^22^ Our study provided an opportunity to examine the different steps conducted as part of an informed consent process, with a particular focus on the quality of written information (i.e., the PICF) and verbal communication (i.e., answering questions, informed consent discussion). The objective of our study was to focus on information accessibility, specifically whether people who had been involved in an informed consent process found information provided in their most recent informed consent experience easy to access, read, and understand. We purposively recruited people for our study from research centers that engage with people with disabilities to ensure that we engaged with communities that are generally under‐represented populations in clinical research.^23^ We partnered with people with lived experience and community leaders across the research life cycle, ensuring the study remained culturally competent and meaningful.

The authors recognize the social and human rights models of disability, where the “disabling” element is caused by the environment and not ascribed to the individual.^24^ Within this context, we use the term “challenge” to refer to the barriers imposed by the environment to information access. For example, an information access challenge occurs when barriers, such as complex language or inaccessible formats, prevent a person from fully engaging with the information. These challenges often arise when there is limited consideration of distinct access needs, whether they relate to vision, hearing, cognition, or language. In this study, we propose that these challenges can be reduced or overcome when research staff employ strategies that actively promote accessibility and inclusion during the informed consent process with potential research participants. The authors, with backgrounds in science, academia, and clinical research, primarily have an outsider perspective. Three of us have been involved in research as participants. Two of us have lived experience of disability and participated as research partners from project planning to publication of findings. This included input into the recruitment plan, invitation materials, survey design, community engagement, and study methods to increase accessibility in study participation.

## STUDY METHODS

### Design and data collection.

This study formed part of a larger participatory research project that involved a wide range of research partners, community members, networks, and consumer and patient advocacy groups. A cross‐sectional survey was developed based on two previously validated surveys developed in the United Kingdom and adapted with permission.^25^, ^26^ The modified survey for the current study contains nine demographic questions and 17 informed consent experience questions. Closed‐ended questions (n = 20) had between three and six multiple choice response options. Open‐ended questions (n = 6) covered topics including motivations for participating in research, perceived positive and negative experiences, and suggestions for how information provision could be improved (see appendix 1, available online; see information about accessing this material in the “Supporting Information” section at the end of this article). The information access scale from the Health Literacy Questionnaire (“reading and understanding information and knowing what to do”), was intended to be embedded into the demographic section.^27^ However, during the survey pilot phase, where survey items were reviewed by stakeholder and consumer partners, the five‐item scale was reduced into one summary literacy indicator item (see appendix 1, item 5).

The survey was prefaced with a short plain language description with a link to review an accessible digital copy in Word document file format of the PICF for participation in our study. The survey was open to adults, some of whom were parents, with appropriate PICF templates used. Information regarding invitation methods and the consent process was provided to referral networks. Study information was provided in varied formats and a link to the Centre for Eye Research Australia's project webpage was also provided where our study's PICF could be accessed, along with a video with open captions that provided an overview of the study (see participant recruitment section below for more details). Survey development followed the principles of “universal design” with the goal of ensuring that it was accessible to people with a range of abilities.^28^ Options to participate in the survey were intentionally varied and included modifying a digital copy online within the Qualtrics platform (more details below) or on paper and administered over the phone. The modifiable digital version was in accessible Word file format and had no locked fields. It could be modified in a way that

**The responsibility and accountability is with research organizations and staff to offer varied information formats to aspire to meet diverse needs.**

was easiest for the respondent to view the material and record their responses. For example, the recipient could adjust the format, magnify the text, and convert the text/background display to suit their preferences. Printed and digital materials were in bold sans serif fonts, sizes 14 for text and 16 for headings, with black print on white. Materials were not routinely provided in braille unless requested. It is recognized that people use multiple methods to access information and may use varied digital technologies depending on the context.^29^

Several digital survey platforms were considered and while there was no single platform that was entirely accessible, Qualtrics software (Qualtrics, Provo, Utah) was selected as the most preferred. This was owing to accessibility for screen readers, high color‐contrast options, enlarged font, and simplified layout. No auditory feedback is possible within Qualtrics, a significant limitation for people that use keyboard shortcuts to navigate and complete online surveys. The built‐in accessibility checker was used, which checks compliance with Web Content Accessibility Guidelines (WCAG) version 2.0 AA recommendations.^30^ Survey items underwent review and modification with research partners^31^ and stakeholders (community leaders and consumer advocates) (n = 9). Flesch‐Kincaid scale readability score of grade three (“relatively simple”) was obtained and the survey re‐piloted (n = 5) with no further changes made. Amendments were approved by a research ethics committee, and the survey was open to respondents from August 2024 to January 2025.

Phone and hard copy responses were manually transcribed into a duplicate survey within the Qualtrics platform. Data from direct and indirect data entry were combined for analysis. Structured phone interviews were not recorded. Survey completion time was estimated to take approximately 15 to 20 minutes. Phone interviews were estimated to take longer, as oral format must first introduce the format of the question, then the question, and then the scale. As such, phone interviews were estimated to take 30 to 45 minutes.

Throughout the study, consultation occurred with experts in the clinical trials sector, leading disability advocates, and community organizations, and with people with varied lived experiences and areas of expertise. Reporting of this study has been guided by the Checklist for Reporting Results of Internet E‐Surveys (CHERRIES),^32^ Standards for Reporting Qualitative Research (SROR),^33^ and the Consensus‐Based Checklist for Reporting of Survey Studies (CROSS).^34^ The study had ethics approval (St. Vincent's Hospital Melbourne HREC: 084/24) and research governance approval (Centre for Eye Research, Australia, July 31, 2024).

### Participant recruitment.

We recruited individuals to participate in our study through national clinical research organizations, clinical trial networks, and disability support service organizations that provide and offer information about research opportunities. Individuals were eligible to participate if they previously had been invited to consider clinical research participation and had undergone an informed consent process (irrespective of their decision to participate or not). All study materials were in English, therefore eligibility to participate required understanding English. The organizations circulated invitations to the study through their research registries and member lists. This purposive sampling method involved direct contact with over 100 Australian research institutes and disability support organizations, across all states and territories. Snowball sampling was also possible with permission for organizations and sites to send on the study invitation to other networks and linked services who participated in research activities. This recruitment method allowed national coverage but precluded the assessment of overall response rate.

Organizations and sites were considered key informants of the communication and information needs of their members/participants. Research staff were encouraged to share invitation materials in ways they perceived to be most effective. Multiple approaches were encouraged, which could include newsletter, social media post, letter, displaying a poster in waiting rooms, and email. Invitation materials contained a plain language summary of the project with a list of the options for accessing further information about the study and ways to complete the survey. The survey could be completed online via the electronic link or QR code, on hard copy with reply paid envelopes supplied to the site, or phone administered where the contact details of the lead researcher were listed. Hard copies of the PICF and survey were supplied to network sites upon request. A digital copy of the PICF could be downloaded from the Qualtrics platform or accessed via the link in the information sheet (which directed individuals to visit the study webpage). The webpage also directed those interested in participating to the online survey platform. For those accessing the survey online, a consent statement was provided, stating that informed implied consent would be obtained if they agreed to proceed to the survey by clicking “survey.” If the survey was administered by phone, verbal consent was obtained and documented. Rather than setting a recruitment target, a six‐month recruitment period was set with an engagement plan to allow equal opportunity across sites to participate.

### Data analysis.

Survey data were exported from Qualtrics to Minitab version 25 (IBM, Armonk, NY) data analysis software. Quantitative data analysis was led by two researchers (FOH, MM). Quantitative data were reported descriptively by count and percentage of the total number of completed surveys. Pearson's chi‐squared test of association was used to determine if there was a relationship across ordinal variables with the probability value (p‐value) reported. Where response category counts were under five, categories were collapsed into best fit categories. If, following this procedure, the count of a category remained under five, the Fisher exact test was reported.

Qualitative content analysis was selected to analyze and interpret the textual content of our qualitative data from open‐ended survey responses.^35^ Data was analyzed to identify patterns and themes, with subsequent interpretation of our findings with existing literature and theory.^21^, ^34^ One researcher (FOH) led the qualitative data analysis, with supervision from and collaboration with two other researchers (TS, CP), each with extensive experience in evaluating qualitative data. NVivo software (QSR, Melbourne) was used to support this analysis. This group undertook a modified Framework Analysis approach.^36^ First, one researcher (FOH) read open‐ended responses to gain familiarity. Second, a coding scheme was derived, driven by deductive content analysis and using the TDF.^21^ The TDF domains provided top‐tier categories for coding data and included skills, beliefs about capabilities, decision processes, environmental context, and resources. Under these categories were second‐order subcategory descriptors, namely barriers to and enablers of information access. Under these subcategories were open codes derived using an inductive content analysis approach.^37^ Subsequent steps involved data excerpts from open‐ended responses being organized under these open codes. After this open coding, the list was synthesized into key themes. During the analysis, researchers acknowledged their own experiences providing informed consent procedures and held each other accountable for the influence they may have had during data analysis.

## STUDY RESULTS

Eighty‐eight people offered implied consent and proceeded to the first item, with 81% (71/88) completing the survey (n.b., percentages henceforth represent N = 71 as the total sample). Survey completion was mainly online (65%), with 20 participants (28%) having their survey completed over the phone as a structured interview, and five participants (7%) completing the survey on paper. Single phone interview sessions ranged from 40 minutes to one hour. The mean age of respondents was 54 (SD 19 years), with an age range between 15 and 94. An even distribution of men and women were involved (48% each; 3% preferred not to indicate and 1% identified as gender diverse), and most (82%) resided in Victoria (Australia). Respondents were required to answer if they identified as having a disability or challenge that affected their ability to receive or express communication and information. Fifty‐two percent reported “yes” to this question. In those willing to describe the impact (92%), this was associated with vision impairment in 34% of participants, and a further 10% described intersectional disabilities. Intersectional disabilities predominantly involved cognitive impairment with or without a learning difficulty. Other areas of neurodiversity were also described (4%). The main motivations for participating in research, noting that more than one reason was often reported, were to help others now and in the future (44%), to contribute to discoveries of potential treatments or a “cure” for their condition (41%), and to advance their own knowledge or medical understanding of their condition (30%).

### Survey quantitative data (N = 71).

Survey participants were asked to reflect on their most recent experience with a research study/trial and the informed consent process for that study/trial. The majority (46%) had undergone an informed consent process within “the last few months.” Respondents described being involved in a range of noninterventional and interventional research covering eye diseases, health conditions, and genomics research. Respondents recalled that information was shared by research staff in a variety of ways and often across verbal and written methods. Most respondents (73%) recalled research staff explaining the research to them verbally. Information was also accessed from reading the information and consent form by themselves (48%) and discussing the study via phone calls and emails (44%). A few respondents (9%) were referred to a website or video for further supplementary information. Most respondents had at least two preferences for reviewing information, with 59% preferring to review and discuss information with a member of the research team, and 44% also opting to review on their own. A quarter of the respondents (25%) also preferred to review information with a family member, carer, or trusted supporter. The majority, 73%, of respondents perceived the time taken by research staff to explain the research with them was “about right.” Almost all respondents were given enough time to decide whether to take part or not (95%).

Most respondents reported that their previous research PICF was easy to read (48%) and easy to understand (52%) (see table 1). In terms of direction, 52% rated the ease of reading the same as ease of understanding, whereas 32% found reading more difficult and 16% found understanding more difficult. Seventy‐seven percent also found their previous research PICF to be “about right” in terms of quantity and length of information. The remaining 23% considered the length of the document “too long.” Twenty‐seven percent of those reporting an information access challenge rated readability of the previous research PICF as hard (Fisher's exact test, p = 0.005, table 1). This may be attributed to the high proportion of respondents who identified with having vision impairment, signifying increased difficulty with reading due to their vision level. Satisfaction levels with the PICF leaned toward being high, either “very satisfied” (23%) or “pretty satisfied” (42%). A smaller proportion was neither satisfied nor dissatisfied (11%) and no respondent reported that they were unsatisfied.

**Table 1:** Informed Consent Experiences by Presence of an Information Access Challenge

	*Access challenge*			
	*No*	*Yes*	*Total*	*p‐value*
	*n = 34 (47.9%)*	*n = 37 (52.1%)*	*n = 71 (100.0%)*	
**Readability of the PICF**				
Hard	0 (0.0%)	8 (26.7%)	8 (14.3%)	0.005
Easy	26 (100.0%)	22 (73.3%)	48 (85.7%)	
**Ease of understanding the PICF**				
Hard	2 (7.7%)	4 (13.3%)	6 (10.7%)	0.675
Easy	24 (92.3%)	26 (86.7%)	50 (89.3%)	
**Satisfaction with the PICF**				
Neither satisfied nor dissatisfied	3 (10.7%)	4 (14.8%)	7 (12.7%)	0.963
Prefer not to say or don't know	1 (3.6%)	1 (3.7%)	2 (3.6%)	
Pretty satisfied	16 (57.1%)	14 (51.9%)	30 (54.5%)	
Very satisfied	8 (28.6%)	8 (29.6%)	16 (29.1%)	
**How much of the PICF was read**				
Did not read it at all	1 (3.7%)	2 (6.9%)	3 (5.4%)	0.592
Don't know or don't remember	3 (11.1%)	2 (6.9%)	5 (8.9%)	
Read about half of it	3 (11.1%)	3 (10.3%)	6 (10.7%)	
Read about a quarter of it	1 (3.7%)	3 (10.3%)	4 (7.1%)	
Read about three quarters of it	2 (7.4%)	6 (20.7%)	8 (14.3%)	
Read every word	17 (63.0%)	13 (44.8%)	30 (53.6%)	
**Literacy indicator**				
Low to moderate	7 (20.6%)	18 (48.6%)	25 (35.2%)	0.013
High	27 (79.4%)	19 (51.4%)	46 (64.8%)	
**Quantity of information in PICF**				
Too much	2 (7.7%)	5 (20.0%)	7 (13.7%)	0.248
About right	24 (92.3%)	20 (80.0%)	44 (86.3%)	
**Length of PICF**				
Too long	(36.0%)	7 (29.2%)	16 (32.7%)	0.610
About right	16 (64.0%)	17 (70.8%)	33 (67.3%)	
**Overall understanding**				
Fairly well	5 (18.5%)	11 (32.4%)	16 (26.2%)	0.222
Well to very well	22 (81.5%)	23 (67.6%)	45 (73.8%)	

Values are frequency (%), p‐values from Pearson's chi‐squared test for literacy, length, and overall understanding, and from Fisher's exact test for all others.

Overall understanding of the previous study they were involved with was mostly rated as “well” or “very well” (31% and 30% respectively). Three respondents rated their understanding as “not well” understood. Fifty‐five percent of respondents perceived that research staff explained the research “very well” and 63% were encouraged to ask questions and felt comfortable doing so. Greater overall understanding of the research study was positively associated with the response to the literacy indicator item (chi‐square test, p = 0.013) but not associated with the quality of the explanation provided by research staff (Fisher's exact test, p = 0.744), nor the satisfaction with the PICF (Fisher's exact test, p = 0.392). As referenced above, a high number of respondents were satisfied with the PICF and explanation by staff (i.e., minimal variation in responses).

### Survey qualitative data (n = 61).

Data were categorized under four domains of the TDF (relevant domains in order of frequency): environmental context and resources, beliefs about capabilities, decision processes, and skills. The number of people reporting a disability or an access challenge that completed open‐ended items (n = 33) and those that did not report an access challenge (n = 29) reported comparable negative and positive experiences regarding information access (questions 18 and 19 respectively). Multiple barriers and enablers to information access were identified and synthesized into five key themes. Theme one was “communication” and was mapped to the TDF domain of skills, representing interpersonal skills and perceived competence of staff. Respondents expressed that optimal communication could be an enabler to information access. If communication was not tailored to their information needs, it could also be perceived as a barrier to information access. There was a trend, in terms of frequency, for respondents with an information access challenge to report face‐to‐face communication of information as a leading enabler (which overlaps with theme four below). Examples of illustrative quotes to highlight the theme as both an enabler and a barrier are shown in table 2 (available online).

Theme two is “PICF quality” and was divided into three sub‐themes: length, language, and format of their previous research PICF. These three subthemes are mapped to the TDF domain of environmental context and resources (all three), and beliefs about capabilities (length). Subthemes are shown in table 2, where quotes to highlight the enablers and barriers associated with accessing information from the PICF are demonstrated.

Theme three is “assistive technology or aides” and is mapped to the TDF domains of environmental context and resources, and beliefs about capabilities. Respondents described a range of methods employed to enable information access. People who identified as having a disability or information access challenge reported a higher number of strategies. Despite their efforts, if information remained inaccessible (i.e., PICF inaccessible with a screen reader), barriers arose (see table 2). Assistive technology and aides included the use of the “read aloud” function within Microsoft documents; computer software enabling text‐to‐speech reading (i.e., screen reader applications); Siri (Apple); verbal dictation; ChatGPT (a large language model web‐based platform, i.e., to aid understanding of language); taking a photo or screen shot to enlarge field; and converting text/background colors (i.e., to white writing on black background for better contrast perception); and enlarging the font or using “zoom text.”

Theme four is “trusted supports to explain information” and is mapped to the TDF domains of social influences, environmental resources, and decision processes. This theme pertained to preferences for information explained verbally to aid decision‐making, particularly if the respondent identified as having an information access challenge associated with vision level. One respondent acknowledged the downside to this situation where the person explaining the information was in control of what information was being shared with them (i.e., loss of autonomy) (see table 2). Respondents advocated for verbal information as a vital alternative to lengthy printed documents, regardless of whether they identified with specific access needs. Furthermore, for those navigating vision‐related barriers, the effort required to make individual adjustments to access information resulted in significant time, effort, mental, and visual fatigue (overlapping with theme three regarding capability and assistive technology literacy and use).

Theme five is “information intervals” and is mapped to the TDF domains of decision processes, and environmental context and resources. This theme relates to information shared at multiple timepoints (i.e., smaller “chunks” of information more often), with continuous information sharing beyond the time when consent was provided to encourage further understanding and absorption of information (see table 2). This included wanting reminders regarding in‐person visit requirements to help them prepare, more structured explanations at each contact point, and being informed of research progress; for example, a newsletter in accessible email format or audio was strongly favored.

Open‐ended survey items offered respondents the opportunity to comment on the perceived positive and negative experiences of receiving information as part of the informed consent process. Importantly, barriers and enablers were reported by people with and without a disability or information access challenge. Generally, concepts expressed overlapped across the cohort. Item 26 in the survey provided an additional opportunity to give feedback on how this process could be improved. Illustrative quotes for future improvements are presented in table 2 for each of the themes described above.

## DISCUSSION

This study explored the experiences of a group of individuals, aged 15 to 91, who had previously been involved with an informed consent process for clinical research. The study considered the opinions and attitudes about their previous informed consent experiences regarding both verbal and written components, through the consent discussion and PICF respectively. A significant proportion of the survey respondents in our study identified as having specific information needs that, if not considered by research staff, made accessing information challenging. Survey participants reported that in their previous experiences with an informed consent process they had to be active in converting communication materials into accessible formats and adopted a range of strategies to do this.

People who identified as having specific information needs reported that they had experienced difficulty in reading a PICF. Ease of reading a PICF was not associated with the quantity or length of information, but rather with the presence of a challenge impacting access to the information. For example, those reporting a vision impairment reported the challenges experienced in trying to read large quantities of information. Some respondents indicated that the effort required to read and comprehend an entire PICF outweighed the perceived benefits. Being provided with a lengthy document, often in small print, is arduous. Previous studies have shown that chronic eye conditions can significantly impact reading speed and ease, with consequential visual and mental fatigue.^38^, ^39^ We found that satisfaction level with a PICF was linked with perceived quantity of information. Of note, two thirds of respondents in this study preferred to review information about research with a member of the research team.

Given the finding that survey participants generally preferred to review information with a member of the research team, it was not surprising that a key enabler for information access was optimal interpersonal and communication skills of the staff of a clinical research study. Many survey respondents described their preference for face‐to‐face or verbal communication to aid understanding of the requirements around research participation. These findings align with previous studies that have shown that good quality extended discussions or “more time talking” is the most effective way to enhance the informed consent process.^5^, ^12^ Videos, audio, and mixed media tools have also been shown to improve understanding.^40^ Yet respondents in our study reported they were not provided with these options when taking part in previous clinical trials or research studies. They recognized, however, that these strategies were possible and were frequently cited as ways to improve the process in the future. Certainly using multimedia information aids in supporting decision‐making for research participation has been established internationally^13^, ^41^ and their use is growing in Australia.^42^, ^43^

Collectively, these findings highlight the need for increased consideration of accessibility and alternative formats to be provided by researchers and research organizations in Australia. The responsibility and accountability is with researchers and research organizations to offer varied information formats to aspire to meet diverse needs. Many of the participants in this study had high literacy levels and have developed strategies to be able to engage with information provided for research participation. But what about those who don't have the skills, capability, motivation, and knowledge to be able to do this? Our study strongly supports the work of others that call for enhancements to equity and inclusion in informed consent practices.^43^, ^44^, ^45^ More broadly, increasing equity and inclusion in health research is predicted to have positive health and economic impacts over the long term.^46^ Our study has implications for policy and practice with the aim of enhancing research participation. In order to reduce current barriers to information access, intentional actions need to be taken by researchers and research organizations early on in the research planning stages.^47^ Adopting implementation strategies around environmental resources, building knowledge and skills capabilities, and understanding the perceived benefits to participants are crucial to offering participant‐focused and informed consent practices. We provide the below recommendations to support these approaches:

**Specific to overall participant engagement principles:**

- Involvement of people with lived experience as research partners to help codesign consent strategies, information materials, and processes.
- Use of accessible language where information is stated plainly and concisely, and medical terms defined.
- Clear explanations and provision of ample time to discuss and answer questions to support shared decision‐making.
- Engagement of trusted supports during the informed consent process.
- Small amounts of information more often (supported by cognitive load theory).^48^, ^49^

**Specific to providing accessible print information, i.e., PICF enhancements:**

- Routine checking of PICF readability scores (i.e., Flesch‐Kincaid scale readability score of grade two to three throughout document).
- Accessible digital formats to allow preferred assistive technology to be used such as screen reader navigation, magnification of text, and text‐to‐speech or speech‐to‐text software.
- Enlarged font, correct use of headers, bullet points in combination with paragraphs to help break up text.
- Shortened disclaimers and legalese sections. Care to not omit crucial information such as the impact to the individual in participating.

**Specific to researcher support and skills capacity:**

- Ensure there is training and support in delivering effective communication. This includes building knowledge and skills around being able to tailor communication to meet a diverse range of participant needs.
- Training and use of multimedia formats to enhance understanding (in line with multimedia learning theories).^50^, ^51^

Our study had strengths and limitations. The data collection method (survey) provided limited context for informed consent procedures that did not involve being provided with a PICF. In instances where people did not receive a PICF in their past research reference study/trial (i.e., verbal discussion over the phone and verbal consent given), many of the survey items were not applicable. This may account for the small number of respondents who started the survey but did not complete it. Online responses could not be verified, and responses overall were subject to recall bias and what respondents were willing to share.

Additionally, the survey did not take an in‐depth exploration of the methods and steps that people might take to access information. Item 6 asked for a description of the impacts to accessing communication information. It tapped into a scale of intrapersonal empowerment,^52^ but arguably was angled toward uncovering challenges rather than being from a strengths‐based approach.

Since all study materials were in English and participants were required to understand English, our results are limited. Importantly, our findings do not provide insights pertaining to culturally and linguistically diverse communities, a group under‐represented in clinical research.

Although we contacted many networks and sites to boost study recruitment, a relatively small number of people participated in our study. This aligns with trends in health and medical research where the broad reach of self‐completed surveys is often offset by lower response rates compared to interviewer‐led approaches.^53^, ^54^ Response rates can be impacted by survey mode, survey length, and population engagement.^53^, ^55^ Directly and actively communicating with the intended population yields a higher response rate.^55^ Reflecting on this, the most effective way to recruit participants for our study was via engagement through disability support organization newsletters and through direct invitation either by phone or email. The latter was leveraged from existing relationships built over time through other research projects, which reaffirms the knowledge base that researchers need to develop relational accessibility, and build trust in the process.^56^ Asking a person what methods to complete the survey were best for them, and telling them what adaptations were on offer, was key to ensuring that the survey was tailorable and accessible. Adapting our survey following varied consumer and consumer representative feedback, and offering survey participation in a variety of modes, are strengths of this study.

The sample size was small and thus limits the generalizability of the findings. Additionally, the study participants comprised a high representation of people who identified with vision impairment. This too limits the generalizability, with under‐representation of other causes for a print disability or information access challenge. However, in Australia, around 18% of the population encounters print disability with unmet vision needs being a leading cause.^57^ A high representation of the cohort citing vision support needs may have been due to any combination of factors including the engagement level of smaller community support organizations compared to larger research institutes. Related are possible differences in resources (e.g., funds and time) available for study invitation and referral. We relied on self‐reporting of an information access challenge. Whether people felt comfortable to disclose/not disclose this information may have influenced the direction of the data. These results are not representative of the wider low‐vision and blindness community. It is very likely that individuals may have experienced barriers to completing the survey despite our flexible methods in accessing the survey, depending upon how the survey invitation materials were disseminated at network organizations. While inclusive guidance (for example references^56^, ^58^) were incorporated throughout, these methodological limitations provide critical lessons for future research.

## CONCLUSION

This study provides insights into some of the strengths and challenges experienced by individuals involved in informed consent processes for clinical research studies. We have highlighted the responsibility of researchers and research organizations to provide accessible communication and information formats. A culture of inclusion is fostered by involving people from the priority population early in research planning and having a set of actions that show intentional consideration of diverse needs. To ensure inclusivity, it is important to ensure that all forms of communication and information are routinely provided in multiple accessible mediums and formats. Sharing effective communication and creating accessible information formats to facilitate informed consent practices are learned skills; therefore, our study highlights the need to invest in supporting researchers to build capacity and capability in these areas. Ultimately, prioritizing accessibility of information and communication will strengthen equity and empowerment in informed consent practices.

## ACKNOWLEDGMENTS

Our deepest gratitude to the participants in this study for their willingness to share their valuable insights, lived experiences, and expert knowledge. We would like to acknowledge the valuable contributions with participant engagement for this study by staff members within Cerulea Clinical Trials, Vision Australia, Blind Citizens Australia, Staphylococcus Aureus Network Adaptive Platform (SNAP) Trial clinical sites and Consumer Reference Group, the Centre for Accessibility Australia, Lions Vision Trials (formally Lions Eye Institute), Australian Clinical Trials Alliance, ARCS Australia, Next Sense, AccessCR, Information Alternatives, VCCC Alliance, Bellberry Limited, Peter MacCallum Cancer Centre, Save Sight Institute, Queensland Eye Institute, and Australasian College of Emergency Medicine.

Data from this study will be made publicly available on the University of Melbourne's Minerva Access and Melbourne Figshare repositories.

## Supporting information

The appendix and table 2 are available in the “Supporting Information” section for the online version of this article and via *Ethics & Human Research*'s “Supporting Information” page: https://www.thehastingscenter.org/supporting-information-ehr/.

Supporting Information

## References

[1] Kadam RA . Informed consent process: A step further towards making it meaningful! Perspect Clin Res. 2017;8(3):107–112. doi:10.4103/picr.PICR_147_16 28828304 PMC5543760

[2] Land V , Parry R , Seymour J , et al. Communication practices that encourage and constrain shared decision making in health-care encounters: systematic review of conversation analytic research. Health Expect. 2017;20(6):1228–1247. doi:10.1111/hex.12557 28520201 PMC5690232

[3] ICH.org . The International Council for Harmonisation (ICH) harmonised guideline for Good Clinical Practice (GCP) E6(R3). Adopted January 6, 2025. https://database.ich.org/sites/default/files/ICH_E6%28R3%29_Step4_FinalGuideline_2025_0106.pdf

[4] Antoniou EE , Draper H , Reed K , et al. An empirical study on the preferred size of the participant information sheet in research. J Med Ethics. 2011;37(9):557–562. doi:10.1136/jme.2010.041871 21478421

[5] Nishimura A , Carey J , Erwin PJ , et al. Improving understanding in the research informed consent process: a systematic review of 54 interventions tested in randomized control trials. BMC Med Ethics. 2013;14. doi:10.1186/1472-6939-14-28 PMC3733934 23879694

[6] O'Sullivan L , Sukumar P , Crowley R , et al. Readability and understandability of clinical research patient information leaflets and consent forms in Ireland and the UK: a retrospective quantitative analysis. BMJ Open. 2020;10(9). doi:10.1136/bmjopen-2020-037994 PMC7473620 32883734

[7] Symons T , Davis JS . Creating concise and readable patient information sheets for interventional studies in Australia: are we there yet? Trials. 2022;23(1). doi:10.1186/s13063-022-06712-z PMC9490706 36131293

[8] Terblanche M , Burgess L . Examining the readability of patient-informed consent forms. J Clin Tri. 2010;2:157–162. doi:10.2147/OAJCT.S13608

[9] Feinberg IZ , Gajra A , Hetherington L , et al. Simplifying informed consent as a universal precaution. Sci Rep. 2024;14(1). doi:10.1038/s41598-024-64139-9 PMC11162480 38851754

[10] Manta CJ , Ortiz J , Moulton BW , Sonnad SS . From the patient perspective, consent forms fall short of providing information to guide decision making. J Patient Saf. 2021;17(3):e149–e154. doi:10.1097/PTS.0000000000000310 27490160 PMC5290300

[11] Stunkel L , Benson M , McLellan L , et al. Comprehension and informed consent: assessing the effect of a short consent form. IRB Ethics Hum Res. 2010;32(4):1–9. PMC4819424 20853797

[12] Flory J , Emanuel E . Interventions to improve research participants’ understanding in informed consent for research: a systematic review. JAMA. 2004;292(13):1593–1601. doi:10.1001/jama.292.13.1593 15467062

[13] Synnot A , Ryan R , Prictor M , Fetherstonhaugh D , Parker B . Audio-visual presentation of information for informed consent for participation in clinical trials. Cochrane Database Syst Rev. 2014;2014(5). doi:10.1002/14651858.CD003717.pub3 PMC6599866 24809816

[14] Cragg WJ , Bishop L , Gilberts R , et al. How can we support research participants who stop taking part? Communications guidance developed through public-researcher collaboration. Res Involv Engagem. 2024;10(1). doi:10.1186/s40900-024-00572-4 PMC11025252 38637845

[15] Symons TJ , Straiton N , Gagnon R , et al. Consumer perspectives on simplified, layered consent for a low risk, but complex pragmatic trial. Trials. 2022;23(1). doi:10.1186/s13063-022-07023-z PMC9795139 36578070

[16] Australian-Bureau-of-Statistics . National Health Survey: health literacy. 2019. https://www.abs.gov.au/statistics/health/health-conditions-and-risks/national-health-survey-health-literacy/latest-release

[17] Australian-Bureau-of-Statistics . Disability, ageing and carers, Australia. 2024. https://www.abs.gov.au/statistics/health/disability/disability-ageing-and-carers-australia-summary-findings/latest-release

[18] World-Health-Organisation . Disability fact sheet. 2023. https://www.who.int/news-room/fact-sheets/detail/disability-and-health#

[19] CT:IQ information can be found here: https://ctiq.com.au/what-we-do-1/.

[20] Ong SWX , Lee TC , Fowler RA , et al. Evaluating the impact of a SIMPlified LaYered consent process on recruitment of potential participants to the Staphylococcus aureus Network Adaptive Platform trial: study protocol for a multicentre pragmatic nested randomised clinical trial (SIMPLY-SNAP trial). BMJ Open. 2024;14(1). doi:10.1136/bmjopen-2023-083239 PMC10806654 38238170

[21] Atkins L , Francis J , Islam R , et al. A guide to using the Theoretical Domains Framework of behaviour change to investigate implementation problems. Implement Sci. 2017;12(1). doi:10.1186/s13012-017-0605-9 PMC5480145 28637486

[22] Michie S , van Stralen MM , West R . The behaviour change wheel: a new method for characterising and designing behaviour change interventions. Implement Sci. 2011;6. doi:10.1186/1748-5908-6-42 PMC3096582 21513547

[23] Aiyegbusi OL , Cruz Rivera S , Kamudoni P , et al. Recommendations to promote equity, diversity and inclusion in decentralized clinical trials. Nat Med. 2024;30(11):3075–3084. doi:10.1038/s41591-024-03323-w 39472759

[24] Retief M , Lesosa R . Models of disability: A brief overview. HTS Teol Stud. 2018;74(1). doi:10.4102/hts.v74i1.4738

[25] O'Sullivan L , Feeney L , Crowley RK , et al. An evaluation of the process of informed consent: views from research participants and staff. Trials. 2021;22(1). doi:10.1186/s13063-021-05493-1 PMC8371296 34407858

[26] Taylor HA , Washington D , Wang NY , et al. Randomized comparison of two interventions to enhance understanding during the informed consent process for research. Clin Trials. 2021;18(4):466–476. doi:10.1177/17407745211009529 33892597 PMC10173028

[27] Osborne RH , Batterham RW , Elsworth GR , et al. The grounded psychometric development and initial validation of the Health Literacy Questionnaire (HLQ). BMC Public Health. 2013;13. doi:10.1186/1471-2458-13-658 PMC3718659 23855504

[28] Williams AS , Moore SM . Universal design of research: inclusion of persons with disabilities in mainstream biomedical studies. Sci Trans Med. 2011;3(82). doi:10.1126/scitranslmed.3002133 PMC3320239 21562227

[29] Olkin R . Making research accessible to participants with disabilities. J Multic Couns Dev. 2004;32:332–343.

[30] World-Wide-Web-Consortium . Web Content Accessibility Guidelines (WCAG) 2.0. 2010. https://www.w3.org/TR/WCAG20/

[31] National Health and Medical Research Council . Draft statement on consumer and community involvement in health and medical research. 2025. https://www.thekids.org.au/globalassets/media/documents/draft-statement-on-consumer-and-community-involvement-in-health-and-medical-research-4.pdf

[32] Eysenbach G . Improving the quality of web surveys: the Checklist for Reporting Results of Internet E-Surveys (CHERRIES). J Med Int Res. 2004;6(3). doi:10.2196/jmir.6.3.e34 PMC1550605 15471760

[33] O'Brien BC , Harris IB , Beckman TJ , et al. Standards for reporting qualitative research: a synthesis of recommendations. Acad Med. 2014;89(9):1245–1251. doi:10.1097/ACM.0000000000000388 24979285

[34] Sharma A , Minh Duc NT , Luu Lam Thang T , et al. A consensus-based Checklist for Reporting of Survey Studies (CROSS). J Gen Int Med. 2021;36(10):3179–3187. doi:org/10.1007/s11606-021-06737-1 10.1007/s11606-021-06737-1 PMC8481359 33886027

[35] Elo S , Kyngas H . The qualitative content analysis process. J Adv Nurs. 2008;62(1):107–115. doi:10.1111/j.1365-2648.2007.04569.x 18352969

[36] Ramanadhan S , Revette AC , Lee RM , et al. Pragmatic approaches to analyzing qualitative data for implementation science: an introduction. Implement Sci Commun. 2021;2(1). doi:10.1186/s43058-021-00174-1 PMC8243847 34187595

[37] Fereday J , Muir-Cochrane E . Demonstrating rigor using thematic analysis: A hybrid approach of inductive and deductive coding and theme development. Int J Qual Meth. 2006;5(1):80–92. 10.1177/160940690600500107

[38] Legge GE . Reading digital with low vision. Vis Lang. 2016;50(2):102–125. PMC5726769 29242668

[39] Remillard ET , Koon LM , Mitzner TL , et al. Everyday challenges for individuals aging with vision impairment: technology implications. Geron. 2024;64(6). doi:10.1093/geront/gnad169 PMC11102008 38124344

[40] Gesualdo F , Daverio M , Palazzani L , et al. Digital tools in the informed consent process: a systematic review. BMC Med Ethics. 2021;22(1). doi:10.1186/s12910-021-00585-8 PMC7913441 33639926

[41] Gillies K , Cotton SC , Brehaut JC , et al. Decision aids for people considering taking part in clinical trials. Cochrane Database Syst Rev. 2015;2015(11). doi:10.1002/14651858.CD009736.pub2 PMC8725643 26613337

[42] Chapman N , McWhirter R , Armstrong MK , et al. Self-directed multimedia process for delivering participant informed consent. BMJ Open. 2020;10(7). doi:10.1136/bmjopen-2020-036977 PMC7383955 32713850

[43] McWhirter RE , Eckstein L . Moving forward on consent practices in Australia. J Bio Inq. 2018;15(2):243–257. doi:10.1007/s11673-018-9843-z 29532387

[44] Judd JA , Griffiths K , Bainbridge R , et al. Equity, gender and health: A cross road for health promotion. Heal Prom J Aust. 2020;31(3):336–339. doi:10.1002/hpja.422 32996234

[45] Mishra SR , Tan AC , Waller K , et al. Conceptualizing, operationalizing, and utilizing equity, diversity, and inclusion in clinical trials: a scoping review. J Clin Epid. 2025;179. doi:10.1016/j.jclinepi.2024.111649 39710302

[46] Bibbins-Domingo K , Helman A . Quantifying the potential health and economic impacts of increased trial diversity. In: Improving Representation in Clinical Trials and Research: Building Research Equity for Women and Underrepresented Groups. National Academies Press. 2022: Appendix A. 36137057

[47] Powell BJ , Waltz TJ , Chinman MJ , et al. A refined compilation of implementation strategies: results from the Expert Recommendations for Implementing Change (ERIC) project. Implement Sci. 2015;10. doi:10.1186/s13012-015-0209-1 PMC4328074 25889199

[48] Chandler P , Sweller J . Cognitive load theory and the format of instruction. Cogn Instr. 1991;8:293–332.

[49] Kirschner PA , Sweller J , Kirschner F , et al. From cognitive load theory to collaborative cognitive load theory. Int J Comp Supp Collab Lear. 2018;13(2):213–233. doi:10.1007/s11412-018-9277-y PMC6435105 30996713

[50] Grunwald T , Corsbie-Massay C . Guidelines for cognitively efficient multimedia learning tools: educational strategies, cognitive load, and interface design. Acad Med. 2006;81(3):213–223. doi:10.1097/00001888-200603000-00003 16501261

[51] Mayer RE , Moreno R . Nine ways to reduce cognitive load in multimedia learning. Edul Psych. 2003;38(1):43–52. 10.1207/S15326985EP3801_6

[52] Bolton B , Brookings J . Development of a measure of intrapersonal empowerment. Rehab Psych. 1998;43(2):131–142. 10.1037/0090-5550.43.2.131

[53] Meyer VM , Benjamens S , Moumni E , et al. Global overview of response rates in patient and health care professional surveys in surgery: a systematic review. Ann Surg. 2022;275(1). doi: 10.1097/SLA.0000000000004078 PMC8683255 32649458

[54] Wilson AB , Brooks WS , Edwards DN , et al. Survey response rates in health sciences education research: a 10-year meta-analysis. Anat Sci Edu. 2024;17(1):11–23. doi:10.1002/ase.2345 37850629

[55] Wu M , Zhao K , Fils-Aime F . Response rates of online surveys in published research: a meta analysis. Comput Hum Beh Rep. 2022;7. 10.1016/j.chbr.2022.100206

[56] Watharow A , Wayland S . Making qualitative research inclusive: methodological insights in disability research. Int J Qual Methods. 2022;21. 10.1177/16094069221095316

[57] Round Table . What is a print disability? (Scroll mid-page to view.) Accessed April 23, 2025. https://printdisability.org.

[58] Research for Development Impact Network . Research for all: making development research inclusive of people with disabilities. 2020. https://rdinetwork.org.au/wp-content/uploads/2020/06/RDI-Network-R4All-Accessible-PDF-1.pdf

