# Supplementary material for: Perspectives of Information Access in the Informed Consent Process for Clinical Research Participation in Australia
Source: Ethics Hum Res. 2026 May 4;48(3):27–39. doi: 10.1002/eahr.70011 (PMC13137929; doi:10.1002/eahr.70011)
Supplement: Supplementary file 1 — Supporting Information [file EAHR-48-27-s001.pdf]

E&HR

Perspectives of Information Access in the Informed Consent Process for Clinical Research Participation in Australia

FLEUR O'HARE, LAUREN N. AYTON, DAVID FORAN, CAMILLE PAYNTER, MICHELLE GALLAHER,
KELLY SCHULZ, MYRA B. MCGUINNESS, LAUREN BARINA, STEVEN Y. C. TONG, AND TESSA SAUNDERS

Table 2. Theme (column A), with correponding Theoretical Domains Framework (TDF) domains (column B). Themes could be both enabers (column C) and barriers (column D) to information access, and illustrative quotes are shown. Suggestions for future improvements for each theme is shown in Column E.

E&HR

A. Theme	B. TDF Domain/s	C. Barrier	D. Enabler	E. Future Improvements
**Communication**	**Skills**	Could have had more explanation around the preparation needed (P39).<br><br>Information is more difficult to read and verbal information more difficult to understand. It can be fatiguing, and at times frustrating, or means we actually don't understand everything fully (P70).	Staff were informative and knowledgeable and polite; followed up actions (P14).<br><br>Clear and precise with no waffle or bureaucratic speak (P18).<br><br>Good conversation at the visit, very informative; felt I could ask questions (P32).	Important to have ongoing conversation and further discussion at each contact point (P23).<br><br>I would like it explained better. I would like life science, citizen science, taught, so the research COI landscape is well known to all (P77).
**PICF language**	**Environmental context & resources**	Some words were too medical and not in layman terms. There were a few	Knew what to expect from the information provided (P26).	More plain language in future studies, especially to consumers/patients. Not everything needs to be in high

A. Theme	B. TDF Domain/s	C. Barrier	D. Enabler	E. Future Improvements
		words I needed to look up (P31).<br><br>Sometimes some parts aren't explained well enough (P51).	Gave me a broad scope of the study, had a good understanding (P36).<br>Succinct (P77).	level scientific, academic language all the time (P51).<br><br>Easy-Read language for people with low IQ - these help (P54).
**PICF format**	**Environmental context & resources**	Receiving the consent form where the formatting was wrong, and box to add signature was way out of proportion (too small) (P16).<br><br>Information sheet could have been more accessible and engaging. It was provided in small print,	Any information in textual formats can only be read if electronic, Word, (where) I can use the "Read Aloud" function to read it to me (P9).<br><br>Having docs supplied in Word makis in much easier with tech (P40).	It would have been good to have the key points enlarged, double the size, to know which bits (are the) most important to read (P43).<br><br>Info sheets that are simplified with visuals are helpful. It would also be good to have links to videos or audio commentary, showing what to expect, real live

A. Theme	B. TDF Domain/s	C. Barrier	D. Enabler	E. Future Improvements
		PDF, and it was very dry (P39).	Size of the print is important, I prefer large text (P44).	scenario, what you will go through (P39).
**PICF length**	**Environmental context & resources, beliefs about capabiities.**	Less paperwork. Too much information is too difficult (P41).<br><br>Big amounts of text meant I had to keep revisiting sections to read content again to make sense of what I had read (P69).<br><br>A little bulky but supposed it had the same excessive compliance issues (P18).	Somewhat long but I understand that all details need to be covered and detailed due to the importance of the study (P21).	Try to use simple language and emphasise the benefits of the research. More info on how long and when the sessions are to be scheduled (P46).

A. Theme	B. TDF Domain/s	C. Barrier	D. Enabler	E. Future Improvements
**Assistive technology, or aides to improve accessibilty**	**Environmental context & resources, beliefs about capabilities.**	I find the welcome leaflet way too complicated, difficult to understand. It should be accessible and be able to be read with a screen reader (P65).<br><br>Unable to visually see the text and document (which) increased the inconvenience needing to use assistive technology device to magnify or convert to accessible media. It increased the time to read and process the information (P7).	In terms of support, assistance with communication tools - i.e. verbal dictation tools - someone to be a scribe etc. Programs with ChatGPT to help understand, break down information, tools to help read out information on a screen, read it out aloud. (P54).<br><br>Due to my vision, blow up the text on laptop, and move head around	Having pre-prepared docs in accessible formats as standard protocol; so people don't have to ask; Easy-Read English; accounting for vision difficulties; making sure the documents are compatible with screen readers; if not provided in accessible format, make sure they know they can ask; information across documents in word, PDF, email, web all accessible (P54).

A. Theme	B. TDF Domain/s	C. Barrier	D. Enabler	E. Future Improvements
			sufficiently to fill in the gaps (P36).	
**Trusted supports to explain information**	**Social influences, environmental resources, decision processes.**	I use someone else to read the information out but it depends on what they think are the main points (i.e. less control over the content shared) (P54).	Have someone read the information aloud or have someone with me (P23). Prefer information to be explained to me due to my vision (P34).<br><br>I don't read long documents, that's just me. I would prefer someone give me information verbally, either over the phone or face to face. I use text to speech, like for long emails, but also	I prefer to discuss the information and have key points highlighted that need reading (P45).

A. Theme	B. TDF Domain/s	C. Barrier	D. Enabler	E. Future Improvements
			have my wife read things and we discuss it (P44).	
**Information intervals:** engagement across multiple timepoints	**Decision processes, environmental context & resources.**	I can't be called out of the blue to make immediate decisions, I like the chance to read, understand, investigate and then respond (P76).<br><br>Follow up discussion once information sheet provided could have been helpful. First contact was phone call, but no opportunity to discuss this information at	Receiving the information over the phone, then in digital form, followed by discussion with the team (were positives) (P31).<br><br>Have information given as well as face to face conversation. Using a number of different mediums is good, so that you have a choice to read, talk about it, and go away with the same	Explaining things at each visit is helpful even if you have read the information sheet. Also, not assuming you know what is going to happen if you have been involved in multiple studies (P27).<br><br>Regular updates for the people involved in research is good (P37).<br><br>Key point would be providing some feedback after the visit.

A. Theme	B. TDF Domain/s	C. Barrier	D. Enabler	E. Future Improvements
		the visit. Straight into testing (P39).	information to read again later (P35).	Good to know how the research is going (P39).<br><br>I like to hear outcomes of any research that I am involved with (P64).
